# Lipopolysaccharide-induced chronic inflammation increases female serum gonadotropins and shifts the pituitary transcriptomic landscape

**DOI:** 10.3389/fendo.2023.1279878

**Published:** 2024-01-08

**Authors:** Christopher Garcia, Leandro M. Velez, Naveena Ujagar, Zena Del Mundo, Thu Nguyen, Chelsea Fox, Adam Mark, Kathleen M. Fisch, Mark A. Lawson, Antoni J. Duleba, Marcus M. Seldin, Dequina A. Nicholas

**Affiliations:** ^1^ Department of Molecular Biology and Biochemistry, School of Biological Sciences, University of California Irvine, Irvine, CA, United States; ^2^ Department of Biological Chemistry, University of California Irvine, Irvine, CA, United States; ^3^ Center for Epigenetics and Metabolism, University of California Irvine, Irvine, CA, United States; ^4^ Division of Reproductive Endocrinology and Infertility, Department of Obstetrics and Gynecology, Prisma Health Upstate/University of South Carolina School of Medicine Greenville, Greenville, SC, United States; ^5^ Center for Computational Biology & Bioinformatics, University of California San Diego, La Jolla, CA, United States; ^6^ Department of Obstetrics, Gynecology, and Reproductive Sciences, University of California San Diego, La Jolla, CA, United States

**Keywords:** pituitary, FSH & LH, lipopolysaccharide (LPS), inflammation, estrous, secretion

## Abstract

**Introduction:**

Female reproductive function depends on a choreographed sequence of hormonal secretion and action, where specific stresses such as inflammation exert profound disruptions. Specifically, acute LPS-induced inflammation inhibits gonadotropin production and secretion from the pituitary, thereby impacting the downstream production of sex hormones. These outcomes have only been observed in acute inflammatory stress and little is known about the mechanisms by which chronic inflammation affects reproduction. In this study we seek to understand the chronic effects of LPS on pituitary function and consequent luteinizing and follicle stimulating hormone secretion.

**Methods:**

A chronic inflammatory state was induced in female mice by twice weekly injections with LPS over 6 weeks. Serum gonadotropins were measured and bulk RNAseq was performed on the pituitaries from these mice, along with basic measurements of reproductive biology.

**Results:**

Surprisingly, serum luteinizing and follicle stimulating hormone was not inhibited and instead we found it was increased with repeated LPS treatments.

**Discussion:**

Analysis of bulk RNA-sequencing of murine pituitary revealed paracrine activation of TGFβ pathways as a potential mechanism regulating FSH secretion in response to chronic LPS. These results provide a framework with which to begin dissecting the impacts of chronic inflammation on reproductive physiology.

## 1 Introduction

Women’s reproductive health depends on a dynamic balance of hormones that is regulated by the hypothalamic-pituitary-gonadal (HPG) axis. Neurons in the hypothalamus release gonadotropin-releasing hormone (GnRH) in tightly controlled pulses to coordinate the secretion and production of the gonadotropins, luteinizing hormone (LH) and follicle stimulating hormone (FSH), from anterior pituitary gonadotropes. These gonadotropins, in turn, target the gonads to mediate the production and secretion of sex hormones and the production/maturation of gametes. The imposition of stress on the HPG axis affects fertility and overall reproductive health (1).

External events or conditions that threaten the homeostasis of an organism, commonly referred to as “stressors”, have been well documented to impair reproduction (2–5). Physical and psychological stressors, such as restraint/immobilization, malnutrition, thermal extremes, nociceptive or neuropathic pain, or social stress, induces stress responses that are mediated by the hypothalamic-pituitary-adrenal (HPA) axis (5). Activation of the HPA axis causes the production and secretion of various hormones, particularly cortisol, that inhibit reproduction by acting on each level of the HPG axis (2, 4). Unlike physical and psychological stressors, inflammation is a stressor that is mediated by an immune response, and the relationship between the immune system and stress responses is complex. Generally, stress-induced cortisol is acutely anti-inflammatory. Inhibitory effects on reproduction similar to those induced by the stressors listed above are seen during acute and severe inflammation (6, 7). Advances have been made in understanding nervous system control of immune cells during stress. However these relationships in the context of reproduction are not fully understood (8, 9).

Classic models of inflammation are commonly induced by the bacterial endotoxin lipopolysaccharide (LPS) and consequent activation of toll-like receptor 4 (TLR4) (6). To date, LPS treatments that evaluate reproductive outcomes induced short-term inflammatory responses by either single or multiple low doses (<1mg/kg), where no study has exceeded a week. Despite these variations in methodologies, the LPS treatments had similar inhibitory effects across several animal models at every level of the HPG axis (6, 7). In contrast, chronic LPS exposure in Yangzhou geese resulted in an increase in serum LH and FSH, revealing the potential for chronic stimulation of the immune system to have an unrecognized mechanism of deregulating gonadotropin secretion (10).

Gut dysbiosis is known to disrupt endocrine homeostasis. Dysbiosis is an imbalance of the gut microbiome that can lead to the leakage of LPS into circulation resulting in endotoxemia (11, 12). In humans, blood serum levels of LPS, LPS to high-density lipoprotein (HDL) ratio, and LPS-binding protein (LBP) were all found to be significantly elevated in polycystic ovary syndrome (11). Further, metabolic endotoxemia in mouse models plays a causative role in obesity and insulin resistance, conditions known to impact the HPG axis (13). Clearly, chronic LPS exposure plays a role in reproductive outcomes. However, current experimental models do not address the impact of long-term as opposed to acute exposure of LPS. Therefore, in this study, we sought to establish a model of LPS-induced chronic inflammation to study the unknown mechanisms linking endotoxemia and dysregulated gonadotropin secretion.

In this study, we examined the impact of long-term inflammation induced by LPS on gonadotropin regulation, beyond the effects of acute inflammation previously described. To produce a low-grade chronic inflammatory model, pre-pubertal mice were injected with LPS twice a week for 6 weeks with a dose below the 50% lethal dose of ~25mg per kg body weight (14). As opposed to adult reproductively mature mice, beginning LPS treatments pre-puberty tests the impacts of inflammatory stimulus on reproductive maturation, a process which can permanently impact adulty fertility. Overall, this model stands in contrast to previous studies evaluating the acute effects of inflammation wherein animal models were injected with either a single sublethal dose or multiple sublethal doses over a week. We demonstrate that serum LH and FSH increased in response to LPS. To investigate the effects of LPS on LH and FSH secretion and production in gonadotropes, bulk RNA-sequencing (RNAseq) was conducted on the murine pituitaries. Our overall approach to analyzing these data was first to assess global changes in gene expression, followed by construction of gene networks to identify important genes and pathways and lastly to use secretome analysis for discovery of potential paracrine mechanisms that regulate gonadotropin secretion. We show that contrary to expectations, genes involved in cell division were induced while genes involved in ribosomal activity were suppressed. In addition, WGCNA analyses identified 2 clusters of coregulated genes that in turn correlated with LPS dosage and gonadotropin levels. Overrepresentation analyses of these clusters revealed enrichment in metabolism and intracellular receptor signaling. Finally, secretome analysis suggests a role for TGFβ2 and Tgfbr3 as a potential factors regulating FSH secretion, thus highlighting a distinct pathway to further interrogate for understanding the impact of chronic inflammation on the female reproductive axis.

## 2 Materials and methods

### 2.1 Animals and LPS treatment

Female C57BL/6N (Harlan Laboratories) mice were housed on a 12L:12D cycle with food and water available ad libitum. Mice were housed at four females per cage. All the experiments were approved by the University of California San Diego and University of California Irvine Institutional Animal Care and Use Committees.

Prepubertal (4 wks of age) females were randomly assigned to one of 4 groups, placebo control (phosphate buffered saline), low dose LPS (lo, 5ng/kg), medium dose LPS (med, 500ng/kg), or high dose LPS (hi, 50μg/kg). Mice were injected intraperitoneally (i.p.) twice-weekly on Monday and Thursday for 6 weeks. After the final injection, mice were euthanized once they reached diestrous (*n* = 8 mice). Additional cohorts for validation studies included the PBS and LPS hi dose groups (at least *n*=3).

### 2.2 Estrous cycle assessment

Estrous cycle stage was determined by light microscopic analysis of smears from vaginal lavage obtained during the course of the 6 weeks beginning at vaginal opening. Vaginal opening was determined by visual examination of the vulva described by Caligioni (15). Proestrus was defined by the presence of mostly nucleated and some cornified endothelial cells, estrus as mostly cornified cells, metestrus as some cornified endothelial cells and mostly leukocytes, and diestrus as primarily leukocytes.

### 2.3 Tissue collection

After 6 weeks of LPS injections, mice were anesthetized with isoflurane, weighed, blood collected via retro-orbital bleeding (cohort 1 with 4 doses), and then rapidly decapitated (between 1000 and 1200 hr) or trunk blood was collected post decapitation (all additional cohorts). Pituitaries were collected, frozen on dry ice, and stored at −80°C. Additionally, dissected ovaries were weighed and stored in RNAlater (Life Technologies) at −80°C until processing for quantitative PCR. Approximately 20μL of blood was collected from mice via lateral tail vein blood every Monday before LPS injection for 3 weeks. Cycle stage was monitored throughout. Serum was separated by centrifugation after allowing blood to clot at room temperature for 1 hr (2,000 x *g* for 10 min at 4°C) and stored in -80°C until assayed.

### 2.4 Hormone and inflammatory marker assays

Blood samples were collected by tail vein or at time of euthanasia, allowed to clot at room temperature for 1 h, centrifuged at 2000×g for 15 min, and then serum was collected and stored at −20°C until assayed for LH and FSH by The University of Virginia (UVA) Center for Research in Reproduction Ligand Assay and Analysis Core or in house via Luminex. Serum T was measured with radioimmunoassay (range 5.0–1075 ng/dl) at the UVA Ligand Core. LH at the UVA Ligand Core was measured using LH RIA with a reportable range between 0.02 and 75.0 ng/mL (intra-assay CV = 5.5%, inter-assay CV = 8.4%). LH is measured in serum by a sensitive two-site sandwich immunoassay (16, 17) using monoclonal antibodies against bovine LH (no. 581B7) and against the human LH-beta subunit (no. 5303: Medix Kauniainen, Finland) as described previous (16). The tracer antibody, (no. 518B7) is kindly provided by Dr. Janet Roser (18), (Department of Animal Science, University of California, Davis) and iodinated by the chloramine T method and purified on Sephadex G-50 columns. The capture antibody (no. 5303) is biotinylated and immobilized on avidin-coated polystyrene beads (7mm; Epitope Diagnostics, Inc., San Diego, CA). Mouse LH reference prep (AFP5306A; provided by Dr. A.F. Parlow and the National Hormone and Peptide program) is used as standard. The assay has a sensitivity of 0.04 ng/ml. Serum and tissue culture LH and FSH were measured by (standard curve range: 4.9-20,000pg/mL and 24.4-100,000 pg/mL respectively) MILLIPLEX^®^ MAP Mouse Pituitary Magnetic Bead Panel (Millipore Sigma, MPTMAG-49K) using a xMAP INTELLIFLEX^®^ Systems (Luminex). TGFβ in conditioned media was measured with the MILLIPLEX MAP TGFβ Magnetic Bead 3 Plex Kit (TGFBMAG-64K-03) according to protocol. Data are represented as mean or linear regression ± SEM. Serum C reactive peptide was measured with the Mouse CRP ELISA from ThermoFisher Scientific (Catalog # EM20RBX10). Serum endotoxin levels were measured (reportable range 0.01 to 0.1 EU/mL, 0.1 to 1.0 EU/mL) with a Pierce™ Chromogenic Endotoxin Quant Kit (Thermo Scientific). Data are represented as mean fold change compared with control ± SEM.

### 2.5 Quantitative PCR of LBT2 cells and ovaries

LβT2 cells were treated with LPS [10ug/mL] for a 24 hrs and/or GnRH [10nM] for 30 min. Ovaries were snap frozen in liquid nitrogen. RNA was isolated using RNeasy Mini kit (Qiagen). Complementary DNA was made by reverse transcription of 1 μg total RNA using High-Capacity cDNA Reverse Transcription Kit (Applied Biosystems). Complementary DNA products were detected using iQ SYBR Green Supermix (Bio-Rad Laboratories) on a CFX Opus 384 Real-Time PCR System. Data were analyzed by the 2ΔΔCt method by normalizing genes of interest (**Table 1**) to *Gapdh* for LβT2 cells or to *Rpl19* for ovaries. Data are represented as mean fold change compared with control ± SEM.

**Table 1:** Real time PCR primer sequences.

Gene Symbol	RefSeq	Primer (5′-3′)	Reverse Primer (5′-3′)
*Gapdh*	NM_001289726.1	TGCACCACCAC CTGCTTAG	GGATGCAGG GATGATGTTC
*Egr1*	NM_007913.5	ATTTTTCCTGAG CCCCAAAGC	ATGGGAACCTG GAAACCACC
*Fos*	NM_010234.2	GGCAAAGTAGAG CAGCTATCTCCT	TCAGCTCCCTC CTCCGATTC
*Cga*	NM_009889.2	CCCCTCAGATCGA CAATCACC	AACATGGACAG CATGACCAGAA
*Lhb*	NM_008497.2	TGTCCTAGCATGG TCCGAGT	CCCCCACAGT CAGAGCTACT
*Fshb*	NM_008045.3	TGACTGCACAGG ACGTAG	TCTACTGAGA TGGTGATGTTG
*Gh*	NM_008117.3	CCTCAGCAGGAT TTTCACCA	CTTGAGGATC TGCCCAACAC
*Tshb*	NM_009432.2	AAGCAGCATCCTT TTGTATTCCC	CCTGGTATTTC CACCGTTCTG
*Txnip*	NM_001009935.2	GGACTACTTGCG CTATGAAG	TTCACCCAGT AGTCTACGCA
*Bbof1*	NM_028377.3	GAAAAGCACCGTT TGGAGCA	GTATGCAAGCG CTTGTGAA
*Gpr82*	NM_175669.4	AAAAGGCTGGC CTCTGGATT	TGCTGGTAGCT CACAGTAGG
*Rpl8*	NM_012053.2	AGCGGACAGAGC TGTTCATC	GATCGTACCC TCAGGCATGG
*Rpl18a*	NM_029751.4	CCAAAATGCCACA CACCACC	CACCTGTCCG CAGTACACAA
*Cyp17a1*	NM_007809	TGGAGGCCACTAT CCGAGAA	CACATGTGTG TCCTTCGGGA
*Cyp19a1*	NM_007810	AGCATTGTGATTGT TCCTCTGG	GGGAGGCTCAG GTTCTGTTC
*Star*	NM_011485	GAACGGGGACGA AGTGCTA	TCCATGCGGT CCACAAGTTC
*Rpl19*	NM_001159483	TTTTGCCCGACGA AAGGGTA	AGCTTCCTGA TCTGCTGACG

### 2.6 Bulk RNAseq of pituitary mRNA

Four individual snap frozen pituitaries from mice representing the median LH response to LPS at each dose underwent total mRNA isolation using the Qiagen RNeasy micro kit with on column DNA digestion according to manufacturer’s protocol. Following Ribodepletion and cDNA library prep with Illumina Total RNA Prep, sequencing was performed on the NovaSeq4 platform. Primary analysis was performed using bcbio-nextgen (19) version 1.2.3. Quality control of the raw fastq files was performed using the software tool FastQC1 v0.11.8. Sequencing reads were trimmed with cutadapt (20) v2.10 and aligned to the mouse genome (mm10,seq,ucsc-201112) using the STAR aligner (21) v2.6.1d. Read quantification was performed with kallisto (22) version 0.44.0 using the mm10 annotation (2018-10-10_92).

### 2.7 Primary pituitary culture

Whole pituitaries were dissected from wild-type C57BL/6 female mice at 9–10 weeks of age. Whole pituitaries were isolated into ice-cold PBS and then dispersed by incubation with 0.25% collagenase Type IV and 0.25% trypsin–EDTA (1x) (Life Technologies) as previously described (23). For immune depletion studies, dispersed pituitary was divided in half, with one half being subjected to depletion of total CD45+ immune cells using Miltenyi Biotech anti-C45 micro beads according to protocol. The cells (dispersed pituitary or immune cell depleted pituitary) were plated on poly-l-lysine (Sigma-Aldrich Inc.) coated Nunc 96-well plates (Thermo Fisher Scientific) at a density of 1.5 × 10^6^ cells per cm^2^. The cells were cultured for 24 h at 37°C and 5% CO2 in high-glucose HEPES-buffered DMEM with 10% FBS prior to experimentation. After pituitary cultures equilibrated they were treated serum starved for 16 hrs, followed by a change in media and 30 min treatment with or without GnRH.

### 2.8 LβT2 cell culture

The female C57BL/6 mouse-derived LβT2 gonadotrope cell line (24, 25) was maintained in high-glucose (4.5 g/l) HEPES-buffered DMEM supplemented with penicillin/streptomycin and 10% fetal bovine serum (FBS: FB-11, Omega Scientific, CA) at 37°C in a humidified atmosphere of 5% CO2. To test the effects of LPS, LβT2 cells were seeded at 2 × 10^5^ cells per cm^2^, cultured for 24 h, and pretreated with serum-free DMEM for 12–16 h prior to LPS treatment.

### 2.9 LPS and GnRH

LPS O111:B4, from Sigma Aldridge was used for all *in vivo* and *in vitro* experiments. LPS was used at a final concentration of 10μg/mL *in vitro* and GnRH (L7134 from Sigma Aldridge) was used at a final concentration of 10nM.

### 2.10 Differential expression analysis

The R BioConductor packages edgeR (26) and limma (27) were used to implement the limma-voom (20) method for differential expression analysis. In brief, lowly expressed genes—those not having counts per million (cpm)³ 1 in at least 3 of the samples—were filtered out and then trimmed mean of M-values (TMM)(19) normalization was applied. After applying a filter for a total sum of counts >10 across all samples and removing transcripts with missing gene names, a total of 14441 genes were obtained and used in subsequent analysis. The experimental design was modeled upon condition and batch (~0 + Treatment). The voom method was employed to model the mean-variance relationship in the log-cpm values, after which lmFit was used to fit per-gene linear models and empirical Bayes moderation was applied with the eBayes function.

### 2.11 Weighted correlation network analysis

A guided walk-through, all scripts and data used to perform WGCNA and trait integration are available at: https://github.com/Leandromvelez/pituitary-LPS-gene-analyses. Briefly, RNA-seq expression data from mouse pituitaries were collapsed into modules using WGCNA (R package), in order to identify clusters of correlated genes. Briefly, goodSamplesGenes function (WGCNA R package) (28) was applied in order to search and then delete low quality data which have too many missing values where all passed initial QC. Therefore, a total of 14441 genes (all genes used for differential expression) were used for module construction. Next, blockwise module construction (blockwiseModules() function) was performed using a minimum and maximum module size of 200, and 2000 genes, respectively, and a standard merge cut height of 0.2 was applied. ME0 was removed, as this module reflects genes whereby WGCNA was unable to assign into specific modules. Further integration with traits data allowed us to obtain regression coefficients and corresponding p-values between module eigengenes and traits, where bicorAndPvalue() function was applied (WGCNA R package). From these module eigengene ~ trait correlations undirected networks were constructed and visualized using qgraph (qgraph R package). A detailed step-by-step analysis, as well as all scripts and data used to perform analysis of DEGs is available at: https://github.com/Leandromvelez/pituitary-LPS-gene-analyses.

### 2.12 Secretome analysis

The list of genes from the processed RNAseq data was reduced to 174 genes that from the mouse genome were predicted or known to be secreted (secretome). We used a published mouse dataset (29) and manual curation (search for families or secreted proteins such as chemokines, cytokines and hormones) to reduce our entire data set to the 174 gene secretome. To reduce the number of genes in this list further, we performed partial least squared discriminate analysis after generating z-scores to identify the top 10% of genes that allow for discrimination amongst treatment groups by genes with the highest variable importance in projection scores. Partial least-squares discriminant analysis and partial least-squares regression analysis are supervised analyses that use linear combinations of variables (treatments groups) to predict the variation in the dependent variables (genes) (30–32). These analytical tools generate principal components (termed latent variables, or LVs) analogous to those obtained by principal component analysis, but constrained by categorical (i.e., PBS, LPS) measures. Variable importance in projection (VIP) analysis combines all LVs over infinite dimensions. A VIP score > 1 is considered important (above average contribution) for model performance only if p < 0.05 in permutation tests that measure variation explained by the model. The genes with the top 15 VIP scores were used to generate a heatmap with hierarchical clustering generated with ClustVis (33). All partial least-squares analyses were conducted in Solo_PLS_Toolbox (Eigenvector Research).

### 2.13 Statistical analysis

All DE, GSEA, GO and co-correlation pairwise p values were subjected to bonferroni corrections to obtain p-adjusted values (see R scripts used for analysis) (34). All qPCR, hormonal, data are expressed as the mean ± SEM for each group. Group differences for all data were analyzed by ANOVA followed by *post hoc* tests of significance as noted in each figure legend appropriate for the experiment. Statistical significance was set at P < 0.05. JMP software was used for statistical testing.

### 2.14 Data and code availability

Murine pituitary RNA-Seq data has been made publicly available via NIH Sequence Read Archive. Further, a detailed walk-through, all scripts used for analysis, as well as all processed data have been made freely available at: https://github.com/Leandromvelez/pituitary-LPS-gene-analyses.

## 3 Results

### 3.1 Chronic low-dose LPS increases serum gonadotropins

**Figure 1:**
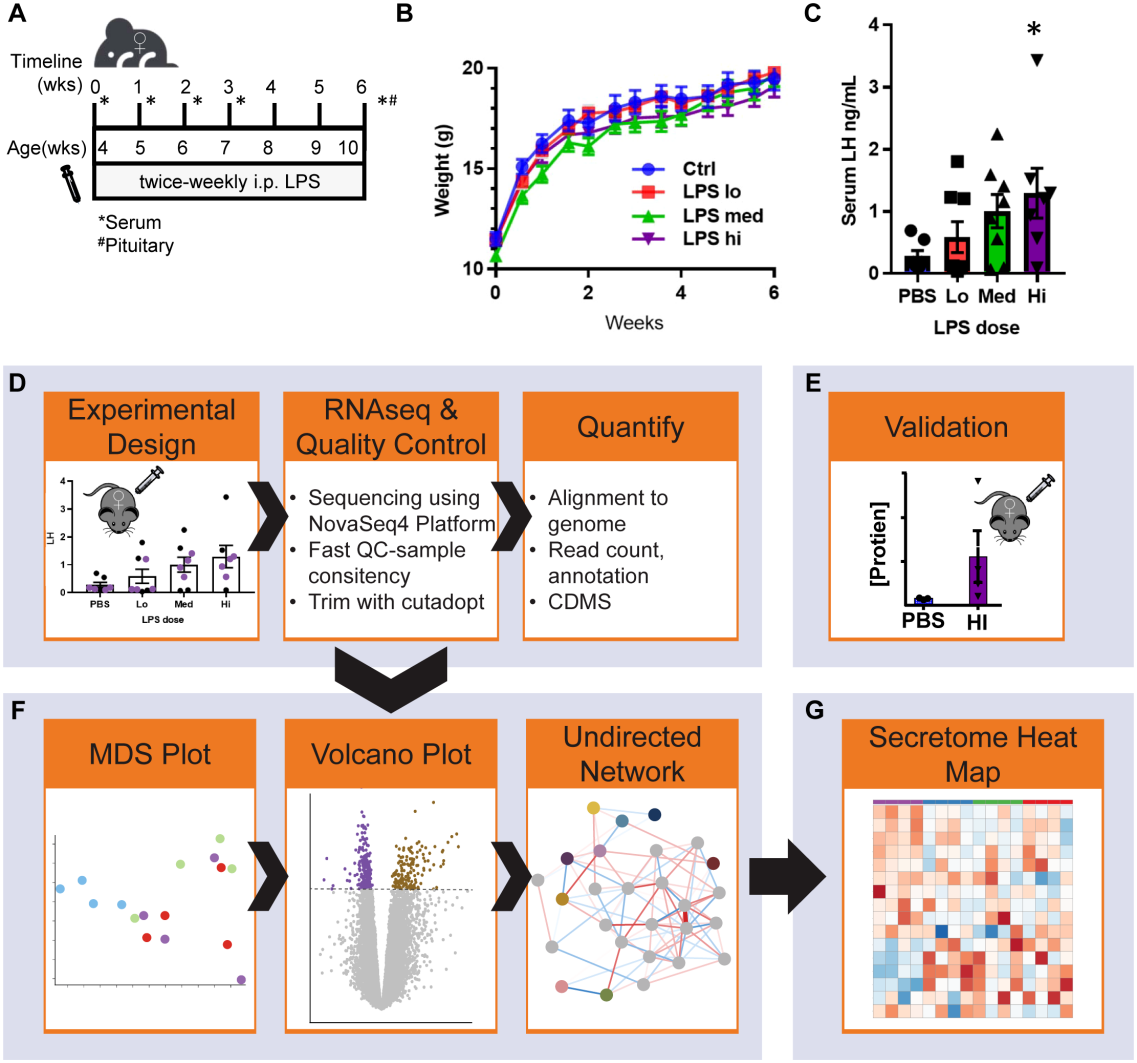
Chronic LPS dose dependently increases serum LH and RNAseq experimental workflow. **(A)** Schematic of experiment design. Female mice 4 weeks of age underwent twice-weekly i.p. injections of PBS (vehicle control) or LPS (Lo 0.005μg/kg, Med 0.5 μg/kg, Hi 50 μg/kg) for 6 weeks. Cycle stage was assessed by vaginal cytology beginning on the day of vaginal opening until first estrous. Mice were euthanized once they reached diestrus following the final injection. Serum and pituitaries were collected for analysis. **(B)** Growth curve of mice over the course of the experiment (*n*=8). **(C)** Serum LH at all doses after week 6 of injections from diestrus staged mice (*n*=8). **(D)** Pituitaries representing the median data points from control and each LPS treatment group were used to isolate mRNA. After generation of cDNA libraries, sequencing was performed. Data was aligned and normalized to counts per million (CPM). **(E)** Serum hormone and physiological validation at Hi dose after 6 weeks of injections. **(F)** Analysis pipeline includes MDS plot from CPM of all samples, followed by differential expression analysis and weighted gene co-expression network analysis WGCNA run in parallel. **(G)** Secretome analysis of the entire dataset. Significance at p<0.05 was determined by ANOVA **(B, C)** and *post-hoc* analysis with Dunnett’s comparison to control test **(C)**.

To determine the impact of low-grade chronic inflammation on female gonadotropin production, we developed a scheme of low dose lipopolysaccharide (LPS) administration (**Figure 1A**). Beginning at 4 weeks of age, mice were injected intraperitoneally with 3 doses of LPS twice-weekly for 6 weeks until they reached sexual maturation. The 50% lethal dose (LD_50_) of intravenous LPS is ~2-26 mg/kg depending on mouse age. Our highest dose, 50μg/kg, is 2 orders of magnitude lower than the LD_50_ of LPS for 10-week-old mice (14). During the course of these injections, there was no difference in weight gain between the experimental and control group (**Figure 1B**) indicating no adverse impact on overall general health and growth that occurs with high doses of LPS (14). We found that serum CRP and endotoxin load from chronic administration of LPS prevented age-associated increases in serum endotoxin, consistent with a state of chronic inflammation and enhanced clearance of LPS from a primed immune system (34) (**Supplementary Figures 1A, B**). Despite acute models supporting an inhibitory role of LPS in gonadotropin secretion, it is known that a positive correlation exists between LPS and LH in women (11). Therefore, we measured serum LH in diestrus staged mice at the end of the six-week LPS injection scheme. Interestingly, LPS increased serum LH in a dose-dependent manner with significance at the highest dose (**Figure 1C**), effectively recapitulating the published relationship of LPS and LH *in vivo*. We conclude that chronic low dose *in vivo* LPS increases serum gonadotropins. Given this conclusion, we designed a workflow to analyze local transcriptomic changes at the level of the pituitary alongside additional evaluation of the reproductive impacts of chronic LPS exposure (**Figures 1D–G**).

**Figure 2:**
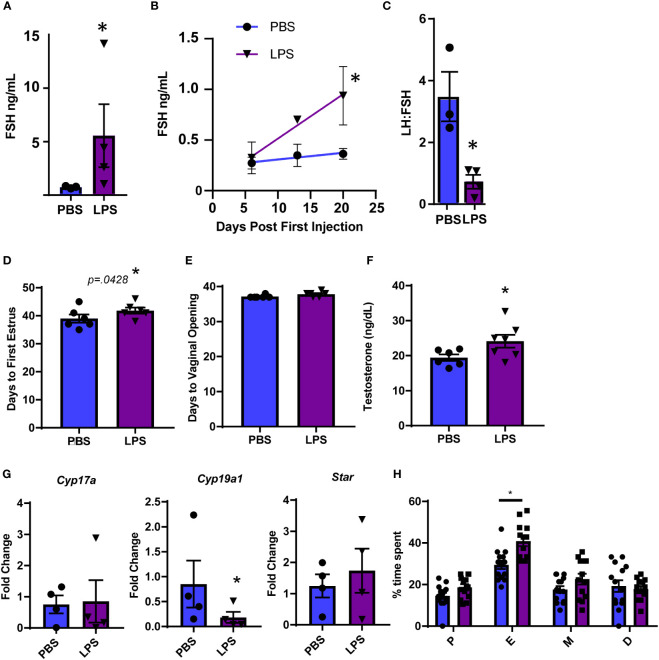
Chronic LPS treatment dysregulates gonadotropins, steroidogenesis and ovarian gene expression. **(A)** Serum FSH from Hi dose LPS treated mice after 6 weeks of injections. **(B)** Serum FSH from 0 to 3 weeks of injections of mice treated with PBS or Hi dose LPS (*n*=2-6 per time point). Only mice in diestrus were included in reported hormones. **(C)** Serum LH to FSH ratio from Hi dose LPS treated mice after 6 weeks of injections. **(D)** Days from birth until vaginal opening of mice treated with PBS or Hi dose LPS (*n*=6). **(E)** Days from birth until first estrus of mice treated with PBS or Hi dose LPS (*n*=6). **(F)** Serum testosterone of mice treated with PBS or Hi dose LPS (*n*=7). **(G)** Fold Change of mRNA transcripts from pituitary of mice treated with PBS and varying doses of LPS (*n*=3-7). All data are mean +/- SEM. Significance at p<0.05 was determined by t-test **(A, C, D–F)** and *Cyp19* and *Star*) or Tukey HSD **(A–C)** or ANOVA (*Cyp17a*) with *post-hoc* analysis with Dunnett’s comparison to control test. Repeated measures ANOVA was used to analyze **(F, H).** The percent of time mice spent in the designated estrous cycles determined by cytology. *n*=14, PBS, 13 LPS. D, diestrus, P, proestrous, E, estrous, M, metestrous. All data are mean +/- SEM. Significance was determined by *student’s t test.* Asterisks indicate significance accepted at p< 0.05 compared to the PBS or LPS control.

To further evaluate hormonal and ovarian disruption in this model, we performed additional experiments at the high dose of LPS given the significance of the increase in LH. First, we measured FSH at the high dose of LPS administration and also found a significant increase (**Figure 2A**). Even more striking is that we found an increase in serum FSH that was apparent by 3 weeks post the first LPS injection (**Figure 2B**). This increase in FSH was the driving factor in a significantly reduced LH to FSH ratio (**Figure 2C**). The significant changes in serum gonadotropin, specifically FSH, indicate that puberty and ovarian maturation may be impacted. Therefore, we tested the impact of our LPS injection scheme on measures of reproductive biology. We found a small, but significant one day delay in time to first estrus, but no significant difference in time to vaginal opening in LPS treated mice (**Figures 2D, E**). We next assessed whether the elevated FSH had an impact on steroidogenesis or ovarian mRNA transcripts. We found that the chronic LPS treatment resulted in elevated serum testosterone (**Figure 2F**). Despite no difference in ovarian weight with increasing doses of LPS, we found that gene expression of *Cyp19* was significantly decreased with LPS treatment while expression of *Cyp17* and *Star* remains unchanged (**Figure 2G**). To stage mice to sac at diestrus, we cycled mice at 9 weeks of age. Analysis of this data showed prolonged estrous consistent with elevated FSH (**Figure 2H**). Overall, from this model, we find that chronic low-level LPS results in increased gonadotropin secretion with downstream impacts on ovaries likely from elevated FSH, though the mechanism is unclear.

### 3.2 LPS suppresses ribosomal pathways while inducing cell division genes in pituitary

To identify potential pathways involved in LPS regulation of gonadotropins in the pituitary, we applied RNA-sequencing (RNAseq) of mRNA extracted from pituitaries of mice treated with and without LPS to probe the transcriptional response to systemic LPS. Specifically, RNA-seq was performed across three LPS doses (4 mice representing the mean of the LH response per dose) (**Figure 1D**). We performed differential expression and network analyses to assess global changes in gene expression (**Figure 1F**), followed by an analysis of the pituitary secretome (**Figure 1G**).

**Figure 3:**
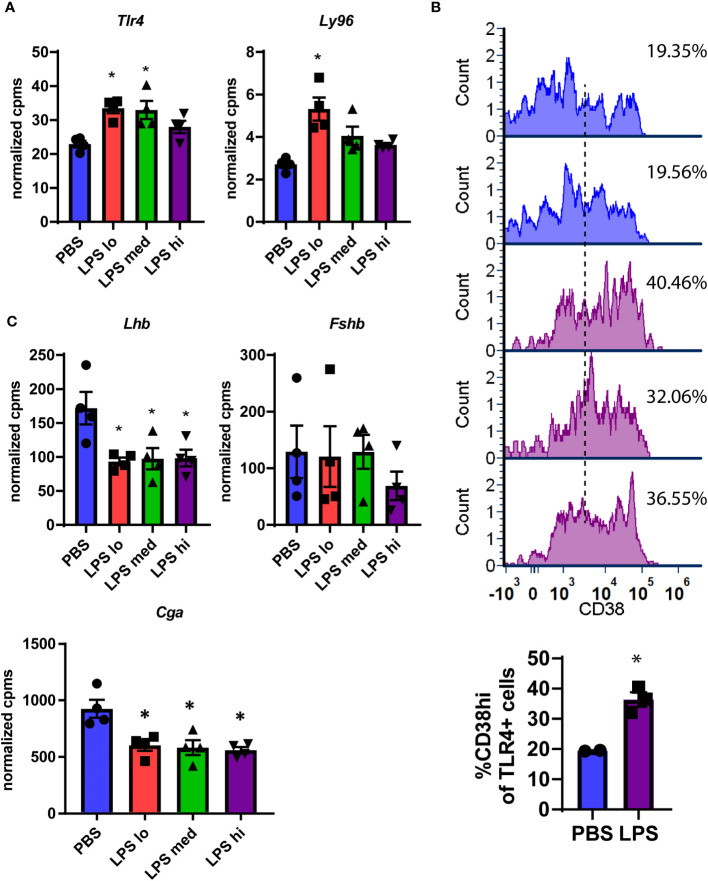
Confirmation of LPS-induced local immune activation. **(A)** Normalized expression data from RNAseq of female mouse pituitary in control conditions (PBS) and under chronic LPS at 3 doses are graphed for *Tlr4* and *Ly96.* **(B)** Flow cytometry analysis of individual pituitary from control mice (PBS) or Hi Dose LPS treated mice. Pituitaries were dissociated and stained for CD45 (immune cells), TLR4, and CD38 (immune activation marker). Histograms are the relative expression of CD38 on CD45+TLR4+ cells (immune cells which express TLR4 protein) normalized to the peak value of each sample. The black dotted line represents the cutoff for high expression of CD38. The inset graph displays the % of TLR4+ cells that are CD38 hi (*n*=2-3) **(C)** Normalized expression data from RNAseq of female mouse pituitary in control conditions (PBS) and under chronic LPS at 3 doses are graphed for *Lhb*, *Fshb*, and *Cga.* Data is mean +/- SEM and was analyzed by one-way ANOVA with a Dunnet’s *post hoc* analysis. Asterisks indicate significance accepted at *p*< 0.05 compared to the PBS control.

First, we analyzed the expression of selected transcripts in the pituitary across the three LPS doses. We confirmed that i.p. LPS reaches the pituitary and has a local effect as evidenced by a significant increase in gene expression for toll-like receptor 4 (*Tlr4*), the natural receptor for LPS, and in *Ly96*, which encodes a coreceptor for TLR4 (**Figure 3A**). This was consistent with increased protein expression of CD38, an immune activation marker, on pituitary immune cells from dissociated pituitaries that express TLR4 protein (**Figure 3B**). Next, we assessed changes in pituitary hormone specific transcripts. In contrast to the elevated LH and FSH measured in the serum, *Lhb and Cga* were significantly decreased at all LPS doses while *Fshb* exhibited no change (**Figure 3C**). Transcripts for all other pituitary hormones including *Prl*, *Pomc*, *Tsh*, and *Gh* were not impacted (**Supplementary Figure 2**). From the specific analysis of these transcripts from the RNAseq data and the flow cytometry data, we can conclude that systemic LPS, either through direct or indirect mechanisms, shifts localized pituitary gene expression which does not correlate with the observed concentrations of serum gonadotropins.

**Figure 4:**
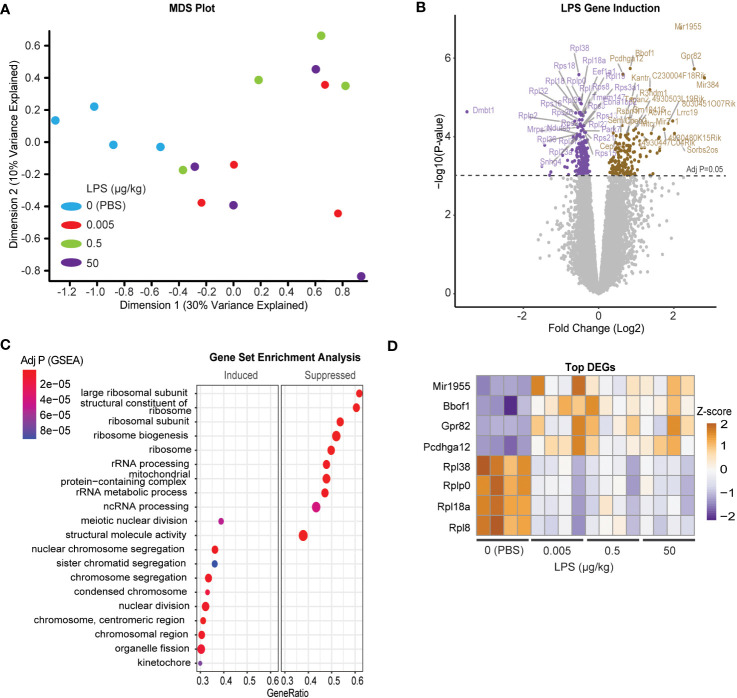
Chronic LPS treatment induces a broad transcriptomic response of cell division and reduced catabolic metabolism in the pituitary of female mice. **(A)** MDS plot from CPM of all samples [Control (blue), or LPS Lo (red), Med (green), Hi (purple)]. **(B)** Volcano plot of differentially expressed genes represented as log fold change of LPS treated over control. **(C)** Gene set enrichment analysis (GSEA) performed on the differentially regulated genes either induced or suppressed in LPS versus CTRL. Significance is indicated as –log10 p value. **(D)** Heatmap of the z-scores of the top LPS-induced and suppressed genes at each dose of LPS. Cut-off p value was set to 0.05.

Due to the incongruency in our data, we used unbiased analysis to understand the global effects of LPS on pituitary gene expression. We performed multiple dimensional scaling (MDS) on the dataset. An MDS plot of the RNA expression data showed distinct clustering between the untreated PBS control and LPS-treated samples (**Figure 4A**). However, the MDS plot did not discriminate between the specific transcriptional differences between LPS doses. Therefore, all LPS-treated samples were grouped for differential expression analysis where 778 total genes were found to be significantly changed at an FDR-adjusted p-value of 1e-3 (**Figure 4B**). Surprisingly, pathway enrichment via gene set enrichment analysis (GSEA) showed that genes encoding ribosomes and ribosomal function were highly suppressed with LPS which is consistent with known impacts on macrophages (35). Meanwhile mediators of cell division processes were strongly induced (**Figure 4C**). Generally, these changes were observed robustly across LPS treatments, regardless of doses used (**Figure 4D**). We, therefore, tested whether direct treatment of LβT2 cells with LPS recapitulated the pituitary RNA changes from *in vivo* administered LPS. We found that 24hr treatment of LβT2 cells with LPS did not significantly regulate the top DEGs or impact the LβT2 cell response to GnRH (**Supplementary Figure 3**). We speculate that some gene changes we see in the pituitary in response to chronic LPS are either not occurring in gonadotropes specifically or are being regulated by indirect or feedback mechanisms through other LPS-responsive cell types such as immune cells or TLR4 expressing cells throughout the HPG axis, for example in the ovary.

### 3.3 WGCNA analysis implicates the engagement of intracellular receptors and metabolism in chronic LPS-induced secretion of LH

**Figure 5:**
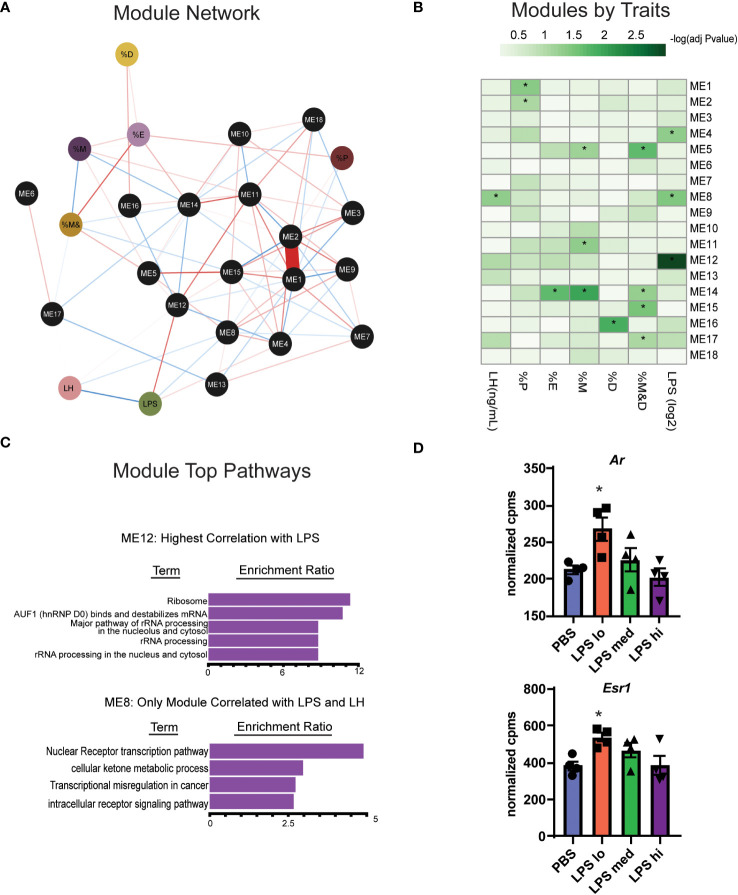
WGCNA analysis identifies two gene clusters associated with chronic LPS induced elevation of serum LH. **(A)** Undirected weighted Network showing association of biological reproductive traits and WGCNA modules. Associations are represented by red (positive association) or blue (negative association) lines and the strength (weight) of the association is represented by the width of the line. Phenotypic traits are in colored circles and Modules (ME) are in black circles. The percent of total time spend in diestrus, proestrus, estrous, and metestrous are represented as %D, %P, %E, and %M respectively. **(B)** Heatmap of correlations among WCGNA module eigengene vectors with reproductive traits. **(C)** Overrepresentation (ORA) analysis of Gene Ontology terms in genes in module 12 and module 8. **(D)** Normalized expression data from RNAseq of female mouse pituitary in control conditions (PBS) and under chronic LPS at 3 doses are graphed for *Ar* and *Esr1.* Data is mean +/- SEM and was analyzed by one-way ANOVA with Dunnet’s *post hoc* analysis. Asterisks indicate significance accepted at *p*< 0.05 compared to the PBS control.

Gonadotropes, the cells which secrete LH, represent at most 10% of the many distinct cell populations in the pituitary that contribute to the transcriptional profiles we observed (23, 36). Furthermore, the transcript for Toll-like receptor 4 (*Tlr4*), the natural receptor for LPS, is significantly increased in response to LPS (**Figure 3A**) and appears present at low levels in many populations of cells found in the pituitary (36). For these reasons, it is difficult to determine which transcripts are important for LPS-induced gonadotropin secretion. To further refine the gene expression response to LPS and incorporate additional relevant physiological outcomes of LPS treatment including serum LH, weighted gene co-expression analysis (WGCNA) (28) was performed on these RNA-seq data to identify sets of specific coregulated gene clusters, termed modules, and determine the relationship among them and additional variables or traits using the reference eigengenes. Visualization of an undirected weighted network of modules and traits highlighted several notable connections. For example, module ME16 and ME14 link estrous cycling parameters with the rest of the network (**Figure 5A**). Additionally, we observed that ME12 and ME8 were linked to LPS or LH. Overall, in several modules we observed significant correlations of genes with specific physiologic outcomes.

One module in particular, ME12, appeared of substantial interest given that it showed the strongest correlation to LPS treatment (**Figure 5B**). ME8, though having a small proportion of DEGs, significantly correlated with both LPS treatment and serum LH. Overrepresentation analysis (ORA) from genes present in ME12 (**Figure 5C**) showed that processes related to ribosome function were strongly correlated to LPS treatment, a similar outcome to the global differential expression analysis performed in **Figures 4B, C**. Moreover, because ME8 was the only module related to both LPS and serum LH, we further analyzed gene expression within these modules to determine relevant processes for the LPS response and LH secretion. Unlike the global pituitary transcriptomic response to LPS, ORA performed with genes from ME8 highlighted metabolic processes and intracellular receptor signaling as important for the outcome of increased LH secretion in response to LPS (**Figure 5C**).

Given the relationship of hormone signaling to metabolism, these analyses suggest that intracellular receptors, such as androgen receptor or estradiol receptor may impact metabolism and act as a potential mechanism linking LPS to elevated serum gonadotropins (23, 37). We analyzed the gene expression of androgen and estrogen receptor alpha (*Ar* and *Esr1*) in the pituitary of LPS treated mice and found that low dose LPS increases the transcript of these intracellular receptors while high dose has similar expression levels as the control (**Figure 5D**). This is consistent with increased testosterone (**Figure 2F**) having negative feedback on AR signaling at the level of the hypothalamus and pituitary (38). Our data suggest that sex steroid feedback loops may be involved in LPS mediated elevation of serum gonadotropins.

### 3.4 Secretome analysis reveals paracrine TGFBR ligands as potential mediators of chronic LPS-induced gonadotropin secretion

**Figure 6:**
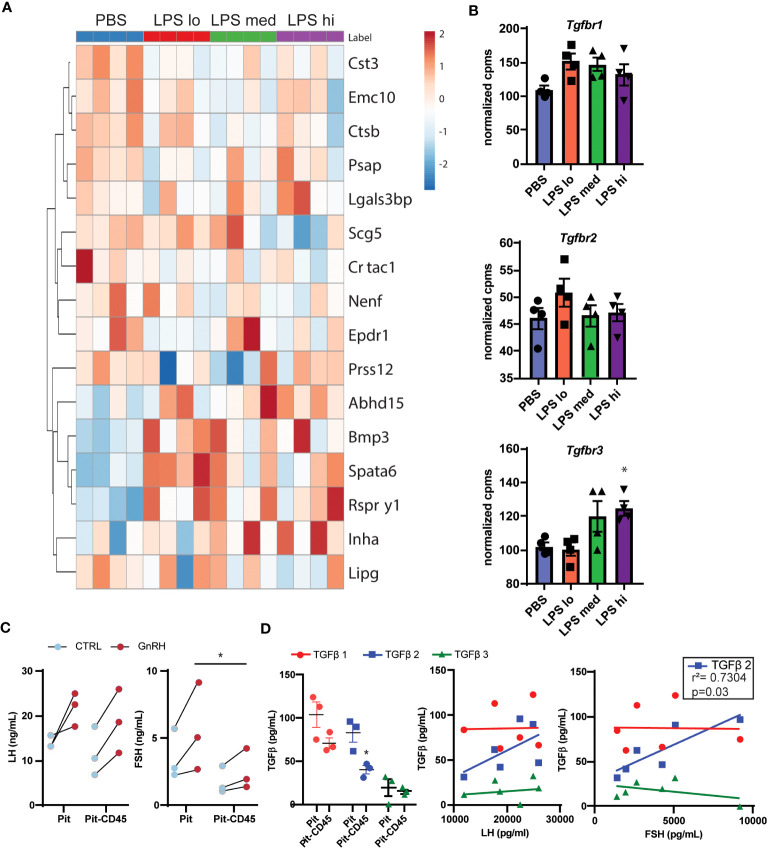
Secretome analysis reveals TGFβ signaling as a potential regulator of LPS-induced FSH secretion. **(A)** Manual curation and a published list of genes in the mouse secretome (39) from the entire RNAseq gene list resulted in 174 identified genes predicted or known to be secreted. The top 16 genes of these 174 that discriminated between LPS treated and PBS in a partial least squares discriminant analysis model are displayed in a heatmap with hierarchical clustering of the genes [Control (blue), or LPS Lo (red), Med (green), Hi (purple)]. **(B)** Normalized expression data from RNAseq of female mouse pituitary in control conditions (PBS) and under chronic LPS at 3 doses are graphed for *Tgfbr1 Tgfbr2*, and *Tgfbr3.* Data is mean +/- SEM and was analyzed by one-way ANOVA with Dunnet’s *post hoc* analysis. Asterisks indicate significance accepted at *p*< 0.05 compared to the PBS control. **(C)** Immune cells from dispersed pituitary of 10-12 week old female mice were cultured for 24 hrs with (Pit) or without (Pit-CD45) endogenous CD45+ immune cells *in vitro*. After overnight serum starvation, the pituitary cultures were stimulated in fresh media +/- 10nM GnRH for 30 min. The conditioned media was analyzed by Luminex and plotted. Each sample represents pituitaries pooled from 3 mice (*n*=3). Data was analyzed by one-way ANOVA. **(D)** Conditioned media from **(C)**, was acid treated to activate TGFβ for analysis by Luminex. TGFβ concentrations are plotted in Pit and Pit-CD45 conditions and correlated to their respective concentrations of LH and FSH (*n*=3). Data is mean +/- SEM and was analyzed by Student’s t test or Pearson correlation. Asterisks indicate significance accepted at *p*< 0.05 compared to the PBS control.

Given the complexity of gonadotropin regulation through secreted factors and receptors, we sought to identify potential paracrine signals in the pituitary involved in this regulation during chronic LPS exposure. First, we used a published mouse secretome (29) and manual curation of our RNAseq dataset to identify 174 genes that were predicted or known to be secreted into the extracellular space. We defined these 174 genes as the secretome. To reduce the number of genes in this list, we performed a partial least squared discriminate analysis to identify the top 10% of genes that allow for discrimination amongst treatment groups by genes with the highest variable importance in projection scores. Using this new list of 16 genes, we created a heatmap and performed hierarchical clustering (**Figure 6A**). One major cluster showed increasing transcripts of genes related to cell metabolism and TGFβ signaling. Specifically, genes encoding bone morphogenic peptide 3 (*Bmp3*) and inhibin A (*Inha*) increase with LPS treatment. Because protein products of these genes bind to TNFβ receptors, we analyzed the transcript levels of *Tnfbr1*, *Tnfbr2*, and *Tnfrb3* in our dataset. We found that *Tnfrb3* was significantly increased at the highest dose of chronic LPS treatment in the pituitaries of female mice (**Figure 6B**). Given these findings, we tested the relationship of local pituitary secreted TGFβ, strong ligands of TGFβR, to LH and FSH. First, we dissociated pituitary (Pit) from 8-10 wk old unstaged female mice. Half of the samples were depleted of CD45+ immune cells (Pit-CD45), a known source of TGFβ. As expected, 30 min GnRH treatment increased the secretion of both LH and FSH (**Figure 6C**). The amount of GnRH-induced secretion of FSH from primary pituitary cultures was significantly reduced when CD45 immune cells were not present. To determine if this outcome coincided with reduced TGFβ secretion, we also measured TGFβ1,2, and 3 in the conditioned media. TGFβ family members were virtually undetectable in basal conditions. However, we found significant concentrations of TGFβ1,2, and 3 in the GnRH-stimulated conditions. CD45 immune cell depletion led to reduced secretion of TGFβ2 (**Figure 6D**). TGFβ2 also significantly correlated with concentrations of FSH but not LH. Given that LPS is well known to induce TGFβ secretion from macrophages, a TLR4 expressing immune cell, we speculate that from these data that paracrine TGFβ2 and non-canonical functions of TGFBR3 are potential contributors to elevated FSH in response to chronic LPS administration.

## 4 Discussion

In this study, we found that low dose chronic LPS results in elevated serum gonadotropins. By performing bulk RNA-seq analysis of pituitary from LPS-treated mice, we show distinct LPS-induced changes in the transcriptomic landscape that correlate with increases in gonadotropin, particularly metabolic and hormone pathways. Further, the broad changes in RNA expression in the pituitary in response to LPS implicate local paracrine mechanisms which may contribute to the effect of LPS-induced immune stress on reproduction. Overall, we provide evidence that TGBβ2 may be an important factor in the observed LPS-induced upregulation of FSH.

Our observation that chronic LPS induces elevated levels of serum LH and FSH stands in stark contrast to the literature’s assessment of high dose and acute LPS impacts on reproduction (4, 5). These short-term studies measured serum LH either over the course of several hours using a sublethal single dose of LPS or at the end of multiple sublethal doses that did not exceed a week. Acute LPS was demonstrated to suppress the HPG axis through impacts on the hypothalamus, and serum LH was found to decrease consistently across various animal models including rats, mice, monkeys, and ewes, despite differences in LPS serotypes and sources (4, 40–42). Some recent studies show increase of serum FSH similar to our study in response to LPS in male mice (43), laying geese (10), and ewes(44) with implications for activation upstream of the pituitary, though the time frames were limited to acute or less than a week.

Several studies highlight discrepancies among transcription, translation, and secretion of gonadotropins (23, 45–50). Our data support this growing literature on the decoupling of regulatory steps that ultimately lead to the production of gonadotropins. In our study, LPS increased serum LH and FSH, yet surprisingly we demonstrated that transcripts for LH and FSH were decreased or did not change, respectively (**Figures 1**, **3**). Another study recently demonstrated this same outcome in birds treated with LPS, that despite reduced pituitary gonadotropin transcript, serum levels of the protein were elevated (10). Our differential expression analysis (**Figure 4**) demonstrates a counterintuitive reduction in transcripts for translational machinery. By means of a WGCNA analysis we identified 2 sets of genes (modules) that correlated with LPS dosage or LH levels, and a further analysis linked these sets of genes with transcriptional regulation pathways and ribosomal and mRNA dynamics, reinforcing the view of the above mentioned post-transcriptional and post-translational control of gonadotropin gene expression by LPS and LH. Together, this dataset implicates post-transcriptional and post-translational control of gonadotropin gene expression such as stabilization of mRNA via RNA binding proteins and stabilization of ribosomal protein (reviewed in (46)). GnRH, the signal that regulates LH secretion, enriches ribonucleoprotein with *Lhb* and *Cga* mRNA (51). Such redistribution of mRNA could support increased LH and FSH translation and ultimately secretion despite a potential overall reduction in ribosomal mRNA or protein.

Another potential point of regulation that addresses the uncoupling of mRNA transcription and translation is mechanisms that induce translation and secretion independent of transcription. The pituitary microenvironment could be a source of such signals. Secretome analysis led to the identification of TGFβ family members. The protein coded by *Bmp3*, upregulated in LPS conditions, is known to induce the transcription of *Inha*, also upregulated with LPS (52) (**Figure 6**). These results would implicate suppression of FSH. However, we see that TGFβ1 and 3 (those with high affinity for TGFBR1 and 2) with known suppressive function do not correlate with FSH in response to GnRH. TGFβ2, on the other hand, not only correlates with FSH, but is low affinity for TGFBR1 and 2. TGFβ2 does bind with high affinity TGFBR3 which is the most highly expressed TGFBR and has plasticity in its regulation of FSH. Conditional gonadotrope knockdown of *Tgfbr3* results in reduced basal FshB and loss of responsiveness to inhibin A (53). Another modality to consider is that TGFBR3, can also be cleaved and function to sequester inhibin A, effectively removing FSH inhibitory signals. This new model of chronic LPS again highlights the heterogeneity and plasticity of pituitary cell types (54) and implicates cellular crosstalk in the regulation of LPS-induced gonadotropin secretion and merit additional studies including single cell-RNA sequencing and single cell functional approaches to uncover new biology of how local pituitary networks regulate gonadotropin secretion.

As reviewed by Bidne et al. (55), our understanding of LPS impacts of endotoxemia on reproduction does not accurately represent physiological conditions such as the temporal pattern of bacterial infection, or ‘leaky gut’, and instead is based on acute high dose exposures. We address the need for more continuous chronic low-level LPS experiments with our model. Our model of LPS differs from majority of the literature in that LPS is administered 1) at a low dose that is well tolerated (**Figures 1B**, **2**) chronically for 6 weeks starting pre-puberty. The timing and dose of LPS administration in our model are likely defining features that differentiate our approach from previously published studies. With chronic exposure, pathways downstream of LPS activation may either become desensitized, similar to tonic exposure of GnRH receptor to GnRH or many other hormone ligand receptor pairs (56). For example, it is well known that immune cells which express TLR4, when re-stimulated with LPS or exposed to chronic LPS become desensitized or tolerant as evidenced by a reduction in glycolysis, reduced capacity to repair tissue damage, and reduced secretion of inflammatory cytokines (39). Though they go unnoticed, there exists a substantial population of immune cells within the murine pituitary, that we have demonstrated are a sizable source of pleiotropic cytokine and that have an impact on FSH secretion (**Figures 6C, D**). Several studies demonstrate that pro-inflammatory cytokines secreted in response to LPS stimulation such as IL-1β have a direct negative effect on the production of gonadotropins, while anti-inflammatory cytokines like IL-10 support reproduction (6, 57, 58). Here we show that TGFβ1,2, and 3 are TGFβR family ligands produced from the pituitary (**Figure 6D**). Inhibin A and bone morphogenic peptide (upregulated with LPS in our dataset) are also ligands of the TGFβR family (**Figure 6A**). There is evidence for both positive and negative regulation of FSH production and secretion when TGFβR is engaged depending on the ligand and the context (52)(59). Changes in the balance of systemic pro-and anti-inflammatory cytokines such as TGFβR ligands in response to chronic LPS could be a potential mechanism by which LPS administration increases gonadotropin secretion.

Our study does have limitations. We draw conclusions based on bulk RNAseq data from a heterogenous tissue. Further validation and single cell approaches will be needed to thorough understand the interaction of soluble mediators and cells in the pituitary microenvironment. Further, the sample size for the computational analysis is limited. WGCNA is most accurate with a large sample size of 50-100. In this study we have 4 animals per group. By using the preliminary conclusions generated from the network analysis we, were able to develop hypotheses which are testable. Most notably, we have identified a potential role for TGFβ2 and TGFBR3 in the mediation of chronic LPS induction of serum FSH. Further, the observed effects on the gonadotropins could be mediated by input and/or feedback from the hypothalamus and or ovary. In short term LPS models, signals from the hypothalamus including pulsatility of GnRH change. It’s possible that changes in pulsatility support the decreased the LH to FSH ratio (**Figure 2**). Given that testosterone is increased in these mice, estradiol may also be impacted and could play a role in yet to be determined impacts of chronic LPS treatment on LH surge and fertility. Finally, TLR4 is expressed in many cell types throughout the HPG axis. A conditional TLR4 KO in gonadotropes, granulosa cells, or other important points of regulation would clarify the mechanisms of LPS action.

Overall, we have created a unique model of chronic inflammation in female mice using serial injections of low dose LPS over 6 weeks. This model has a phenotype of elevated serum LH and more significantly, FSH and has implications for dissecting the impact of endotoxemia on reproduction. Much is left to be discovered using this new model including impacts on sexually mature females such as female sex steroids, fertility, and ovarian function. Of particular interest is the impact of immune crosstalk in the pituitary microenvironment and the mechanisms of regulation upstream of the pituitary in the hypothalamus including Kiss and GnRH neuron function. Our findings here lay the groundwork and rationale for thoroughly evaluating this new mouse model of chronic LPS-induced inflammation on reproductive function and could eventually be the foundation for the design of immunotherapy for reproduction resulting from chronic inflammation.

## Data availability statement

The data presented in the study are deposited in Dryad, DOI: 10.5061/dryad.x69p8czrd, and in Sequence Read Archives, accession number PRJNA1054603.

## Ethics statement

The animal study was approved by University of California Irvine and San Diego Institutional Animal Care and Use Committee. The study was conducted in accordance with the local legislation and institutional requirements.

## Author contributions

CG: Data curation, Formal analysis, Investigation, Visualization, Writing – original draft, Writing – review & editing. LV: Data curation, Formal analysis, Visualization, Writing – original draft, Writing – review & editing. NU: Data curation, Formal analysis, Writing – review & editing, Investigation. ZD: Data curation, Formal analysis, Investigation, Writing – review & editing, Visualization. TN: Data curation, Investigation, Writing – review & editing, Methodology. CF: Data curation, Investigation, Writing – review & editing, Conceptualization. AM: Data curation, Writing – review & editing, Formal analysis, Visualization. KF: Data curation, Formal analysis, Visualization, Writing – review & editing, Investigation, Resources. ML: Resources, Writing – review & editing, Conceptualization. AD: Conceptualization, Resources, Writing – review & editing, Funding acquisition, Investigation, Project administration. MS: Visualization, Writing – original draft, Investigation, Project administration, Resources, Writing – review & editing, Data curation, Formal analysis, Methodology, Supervision. DN: Data curation, Formal analysis, Investigation, Methodology, Project administration, Resources, Supervision, Visualization, Writing – original draft, Writing – review & editing, Funding acquisition.
