# Supplementary material for: Lipopolysaccharide-induced chronic inflammation increases female serum gonadotropins and shifts the pituitary transcriptomic landscape
Source: Front Endocrinol (Lausanne). 2024 Jan 8;14:1279878. doi: 10.3389/fendo.2023.1279878 (PMC10801245; doi:10.3389/fendo.2023.1279878)
Supplement: Supplementary file 1 [file Image_1.pdf]

Castro-Garcia et al.
Supplemental Figure 1

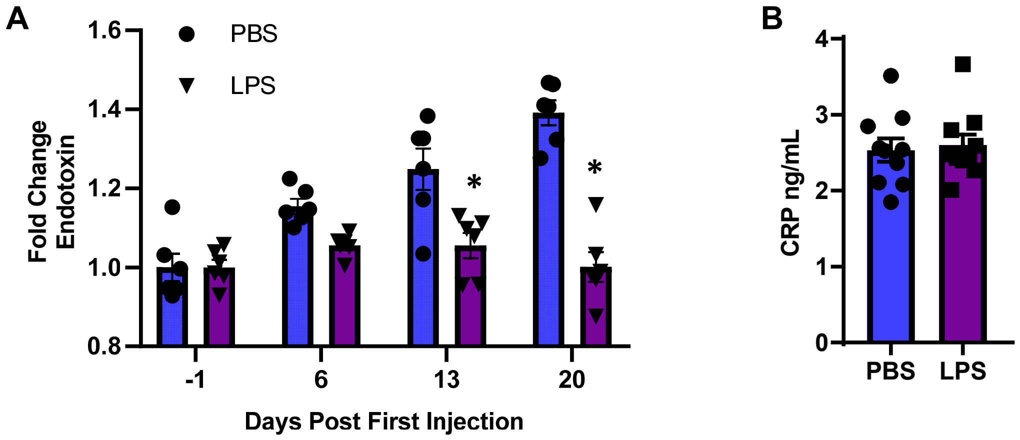

Supplemental Figure 1. Female mice 4 weeks of age underwent bi-weekly i.p. injections of PBS (vehicle control) or LPS for 6 weeks. Cycle stage was assessed by vaginal cytology for 2 weeks beginning after week 4 of i.p. injections. Mice were sac'd once they reached diestrus following the final LPS injection. (**A**) Fold change serum endotoxin. (**B**) Serum C reactive peptide (CRP) in Ctrl and LPS hi dose from staged mice (n=4). All data are mean +/- SEM.
