# Supplementary material for: Lipopolysaccharide-induced chronic inflammation increases female serum gonadotropins and shifts the pituitary transcriptomic landscape
Source: Front Endocrinol (Lausanne). 2024 Jan 8;14:1279878. doi: 10.3389/fendo.2023.1279878 (PMC10801245; doi:10.3389/fendo.2023.1279878)
Supplement: Supplementary file 2 [file Image_2.pdf]

Castro-Garcia et al. Supplemental Figure 2

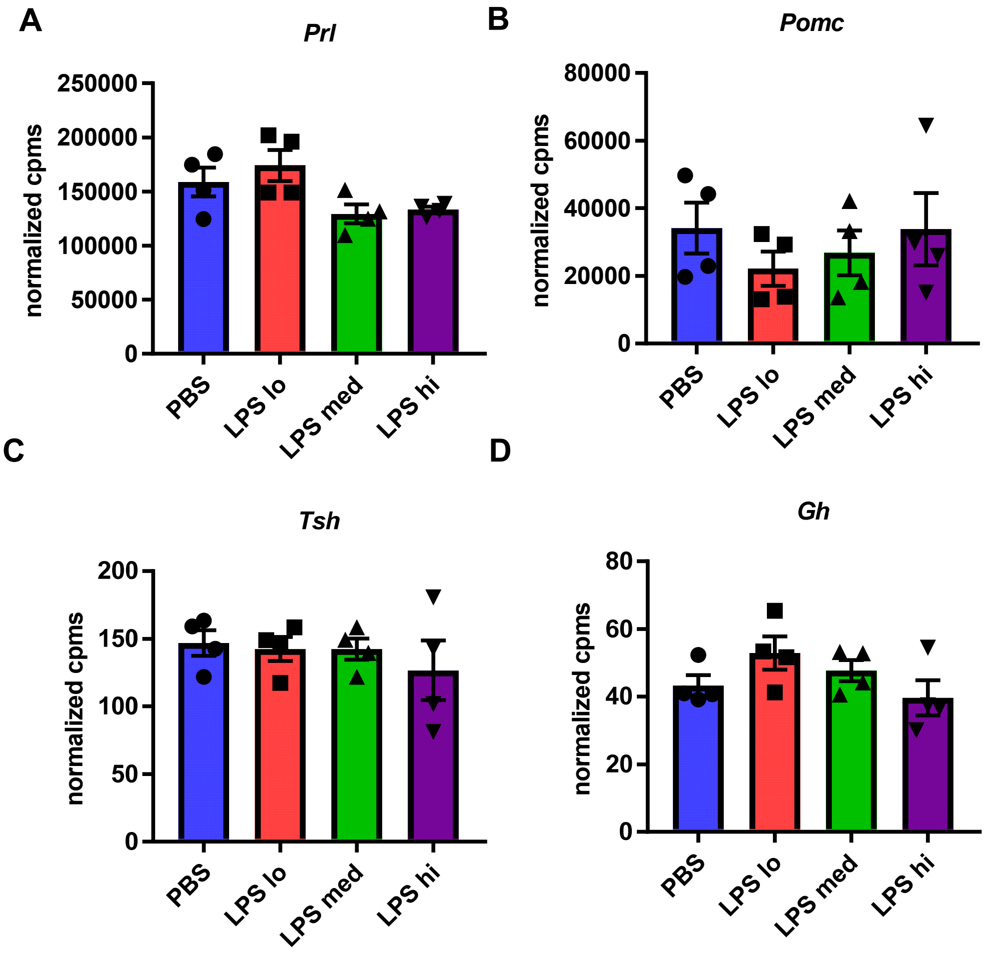

Supplemental Figure 2. Normalized expression data from RNAseq of female mouse pituitary in control conditions (PBS) and under chronic LPS at 3 doses. Data is mean +/- SEM and was analyzed by one-way ANOVA with a Dunnet's post hoc analysis. Asterisks indicate significance accepted at $p < 0.05$ compared to the PBS control.
