# Supplementary material for: Lipopolysaccharide-induced chronic inflammation increases female serum gonadotropins and shifts the pituitary transcriptomic landscape
Source: Front Endocrinol (Lausanne). 2024 Jan 8;14:1279878. doi: 10.3389/fendo.2023.1279878 (PMC10801245; doi:10.3389/fendo.2023.1279878)
Supplement: Supplementary file 3 [file Image_3.pdf]

Castro-Garcia et al.
Supplemental Figure 3

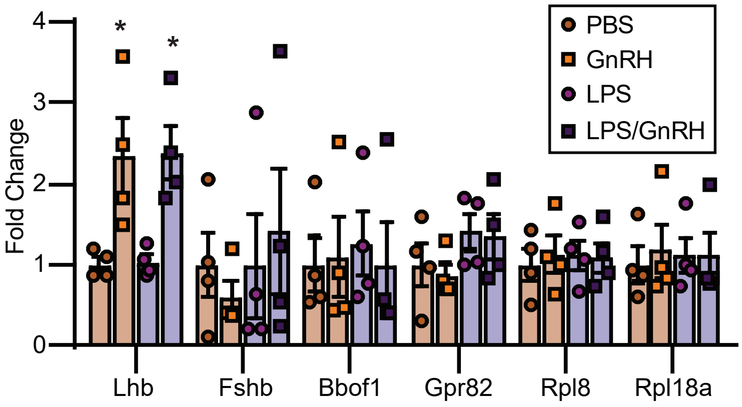

Supplemental Figure 3. Direct treatment of LβT2 cells dσes not recapitulate in vivo LPS induced transcriptomic changes. LβT2 cells were serum starved and treated with or without LPS for 24 hours prior to 30 min GnRH treatment, isolation of mRNA and qPCR analysis. Data is mean +/- SEM and was analyzed by one-way ANOVA with a Dunnet's post hoc analysis. Asterisks indicate significance accepted at $p < 0.05$ compared to the PBS or LPS control.
